# A glycosaminoglycan microarray identifies galectin-4 binding to sulfated glycosaminoglycans

**DOI:** 10.1016/j.bbrep.2026.102689

**Published:** 2026-06-29

**Authors:** Kanae Sano, Meri Nagatomo, Katsunobu Shigematsu, Kazuhiko Yamasaki, Dinh Xuan Tuan Anh, Akira Shibuya, Yuya Otsuka, Toshikazu Minamisawa, Hiroaki Tateno

**Affiliations:** a Cellular and Molecular Biotechnology Research Institute, National Institute of Advanced Industrial Science and Technology (AIST), Central 6, 1-1-1 Higashi, Tsukuba, Ibaraki, 305-8566, Japan; b Central Research Laboratory, Seikagaku Corporation, Higashiyamato-shi, 207-0021, Japan; c Department of Immunology, Institute of Medicine, University of Tsukuba, Tsukuba, Ibaraki, 305-8577, Japan; d Program in Humanics, University of Tsukuba, Tsukuba, Ibaraki, 305-8577, Japan; e Molecular Biosystems Research Institute, National Institute of Advanced Industrial Science and Technology (AIST), 1-1-1 Higashi, Tsukuba, 305-8566, Japan; f Program in Human Biology, School of Integrative and Global Majors, University of Tsukuba, Tsukuba, Ibaraki, 305-8575, Japan; g R&D Center for Innovative Drug Discovery, University of Tsukuba, Tsukuba, Ibaraki, 305-8577, Japan; h Institute of Medicine, University of Tsukuba, Central 6, 1-1-1 Higashi, Tsukuba, Ibaraki, 305-8566, Japan

## Abstract

Lectin–glycosaminoglycan (GAG) interactions remain incompletely understood despite their potential roles in cell signaling and immune regulation. In this study, we performed a comprehensive profiling of the binding specificity of 49 types of human endogenous lectins—including SIGLECs, C-type lectins, and galectins—for GAGs using GAG microarrays. GAGs containing 6-O-sulfation, such as heparin (HP), chondroitin sulfate C (CSC), and chondroitin sulfate E (CSE), exhibited broad binding to multiple SIGLECs and C-type lectins, whereas non-sulfated or low-sulfated GAGs exhibited minimal interactions, indicating a strong dependence on sulfation patterns. In contrast, most galectins displayed little or no detectable binding to GAG. Notably, galectin-4 (Gal-4) uniquely exhibited significant affinity for 6-O-sulfated GAGs, particularly HP. Surface plasmon resonance analysis revealed high-affinity binding of Gal-4 to HP (Kd = 4.70 × 10^−8^ M), substantially stronger than its carbohydrate recognition domains, indicating cooperative contributions of both N- and C-terminal CRDs of Gal-4. Molecular dynamics simulations further supported a binding mode involving both N- and C-terminal domains. Consistent with these findings, Gal-4 bound to endogenous HP-positive mast cells, and this interaction was competitively inhibited by HP. Together, these results identify Gal-4 as a unique galectin with a noncanonical capacity to recognize sulfated GAGs, revealing an alternative glycan-recognition mechanism beyond the conventional β-galactoside paradigm.

## Highlights

• Binding of 49 endogenous lectins to glycosaminoglycans (GAGs) was profiled using a GAG microarray.
• Galectin-4 uniquely shows strong binding to 6-O-sulfated GAGs, particularly heparin, unlike other galectins.
• Galectin-4 detects heparin-positive mast cells in mouse tissues, and this interaction is inhibited by heparin.
• Galectin-4 binds heparin cooperatively via its N- and C-terminal domains.

## 1 Introduction

Glycosaminoglycans (GAGs) are negatively charged, unbranched polysaccharides composed of repeating disaccharide units enriched with sulfate and carboxyl groups [1]. Major classes include heparin (HP), heparan sulfate (HS), chondroitin sulfate (CS), dermatan sulfate (DS), keratan sulfate (KS), and hyaluronan (HA) [1]. Beyond providing structural support in tissues, GAGs regulate cell signaling through interactions with growth factors, cytokines, and receptors [[2], [3], [4]].

Lectins are glycan-binding proteins that recognize specific glycan structures and mediate diverse physiological and pathological processes, including cell–cell communication, signal transduction, immune regulation, pathogen recognition, inflammation, and tissue homeostasis [[5], [6], [7]]. Based on structural features, lectins are classified into multiple families, such as C-type lectins, galectins, and sialic acid–binding immunoglobulin-like lectins (SIGLECs). Humans express a large repertoire of lectins across these families [7]. Glycan ligands of lectins have been characterized using both quantitative and qualitative approaches [[8], [9], [10]]. Extending these platforms, GAG microarrays have been developed to identify proteins that interact with defined GAG motifs, enabling the profiling of growth factors, cytokines, chemokines, extracellular matrix proteins, enzymes, and pathogen-associated proteins [[11], [12], [13], [14]]. We previously established a fluorescence-based GAG microarray for high-sensitivity screening of protein-GAG specificity and applied it to the SARS-CoV-2 spike (S) protein, which exhibited concentration-dependent binding not only to HP/HS but also to chondroitin sulfate E (CSE) [15]. Despite these advances, systematic understanding of interactions between endogenous lectins and GAGs remains limited.

In this study, we employed GAG microarrays to systematically profile the binding of human endogenous lectins to GAGs. While multiple SIGLECs and C-type lectins exhibited broad binding to 6-O-sulfated GAGs, most galectins showed little or no interaction. Notably, galectin-4 (Gal-4) uniquely displayed strong binding to sulfated GAGs.

Gal-4 is a tandem-repeat type galectin predominantly expressed in intestinal epithelial cells and localized both intracellularly and extracellularly, including on the cell surface and in secreted form [16]. It regulates epithelial membrane organization, cell adhesion, and immune responses, particularly in the intestinal tract. Characterizing the binding specificity of Gal-4 toward endogenous glycans is therefore essential for understanding its functions [16]. Here, we further examined the Gal-4–HP interaction using SPR, tissue staining, and MD simulations. Our results demonstrate that Gal-4 exhibits a noncanonical capacity to bind 6-O-sulfated GAGs, revealing a distinct glycan-recognition mode compared with other galectins.

## 2 Materials and methods

### 2.1 GAG

Sources and structures of GAGs used in this study are summarized in Table S1 and Fig. S1. Oxidized CH chondroitin (OxCH) was prepared by selective oxidation of the primary hydroxyl groups of chondroitin (CH), following a report described for the preparation of oxidized HA [17].

### 2.2 Disaccharide composition analysis

GAGs were characterized by conventional disaccharide composition analysis as previously reported [18].

### 2.3 Preparation of GAG–BSA conjugates

GAG–BSA conjugates were prepared using previously reported methods [15]. Briefly, GAG was biotinylated at carboxyl groups, purified by ethanol precipitation, and lyophilized. The biotinylated GAG was thiolated at the reducing end and conjugated to maleimide-modified BSA. After quenching unreacted groups and removing excess reagents, the final conjugate was buffer-exchanged, concentrated, and analyzed by size-exclusion chromatography.

### 2.4 GAG microarray production

GAG microarrays were produced as previously described [15]. In brief, the GAG-BSA conjugate was dissolved in spotting solution and spotted in triplicate onto epoxy resin-coated glass slides using a non-contact microarray printer. The slides were then incubated at 25°C for 3 h for immobilization. After washing with probing buffer, the slides were blocked with TBS containing 1% BSA for 1 h at room temperature.

### 2.5 Preparation of endogenous lectins

The carbohydrate recognition domain (CRD) or extracellular domain of C-type lectins and SIGLECs was cloned into the pSecTag2 vector or pcDNA5/FRT containing a human IgG1 Fc region at the C-terminus to generate Fc-fusion proteins. The expression vector was transiently transfected into HEK293T cells, and the supernatant containing the Fc-fusion protein was purified using Protein G-Sepharose 4 Fast Flow (GE Healthcare). The full-length, N- or C-terminal domains of galectins were cloned into pET vectors (11a, 27b, or 29b), expressed in *E.coli*, and purified by lactose-Sepharose CL-4B (GE Healthcare) [14]. The purity of the lectins was analyzed using sodium dodecyl sulfate–polyacrylamide gel electrophoresis (SDS-PAGE) and western blotting. The protein concentration was calculated using the bicinchoninic acid (BCA) protein assay kit (Thermo Fisher Scientific). Gal-4, Gal-9, P-selectin, E-selectin, and SIGLEC1 were purchased from ACROBiosystems (Tokyo, Japan). Endogenous lectins used in this study were shown in Table S2.

### 2.6 GAG microarray analysis

Galectins (10 μg) were fluorescently labeled with 10 μg of Cy3 Mono-Reactive dye (Cytiva, Tokyo, Japan), and excess Cy3 was removed with Sephadex G-25 desalting columns (Cytiva, Tokyo, Japan). The degree of labeling was calculated from the molar concentration of Cy3 dye [Cy3] divided by the concentration of protein [Protein].

[Protein] = A^corr^_280_ / ε_protein_. A^corr^_280_ = A_280_ − (A_550_ × CF), CF = 0.08, ε_protein_:calculated from the amino acid sequence.

[Cy3] = A_550_ / ε_Cy3,_ ε_Cy3_ = 150,000 M^−1^cm^−1^

Cy3-labeled galectins diluted in the probing solution (25 mM HEPES buffer (pH 7.5) containing 150 mM NaCl and 1% Triton X-100)(10 μg/mL) were applied to each well of the glass slides (80 μL/well) and incubated at 20°C overnight, after which the fluorescent images were acquired immediately using a Bio-Rex scan 200 fluorescence scanner (Rexxam Co. Ltd., Kagawa, Japan).

The Fc fusion proteins of SIGLECs and C-type lectins diluted in the probing solution (10 μg/mL, 80 μL/well) were incubated with each chamber of the glass slides at 20°C overnight. After washing with 100 μL of probing solution twice, each well was incubated with 1 μg/mL of Cy3-labeled goat anti-human IgG Fc (Cat No. 109-165-098, Jackson ImmunoResearch, West Grove, PA, USA). The net intensity value for each spot was determined by signal intensity minus the background value. Data were then analyzed in R (version 4.5.1). The lectin-binding matrix was converted into a numerical matrix with lectins assigned as row identifiers. To reduce the influence of highly abundant signals and improve visualization, binding intensities were transformed using a log2(x + 1) transformation. Heatmaps were generated using the pheatmap package in R. The resulting heatmap was used to visualize lectin-binding patterns across the analyzed samples.

Differences in glycan-binding intensities between Gal-4 and the other galectin family members were assessed using the Wilcoxon rank-sum test. P values were used to determine statistical significance and were annotated as follows: ∗∗∗P < 0.001, ∗∗P < 0.01, ∗P < 0.05, and ns (not significant) when P ≥ 0.05.

### 2.7 Surface plasmon resonance (SPR)

GAG-BSA (5 μg/mL) was immobilized onto a Sensor Chip SA (GE Healthcare) and subsequently exposed to Gal-4 at concentrations of 1.2, 2.5, 5, 10, and 20 × 10^−7^ M, or to Gal-4N/C at concentrations of 2.47, 7.41, 22, 66.7, and 200 × 10^−7^ M. All interactions were analyzed in HBS-P buffer (GE Healthcare) using the Biacore X100 system (GE Healthcare).

### 2.8 Glycoconjugate microarray analysis

Glycoconjugate microarray production and analysis were performed by following the previous report [19]. Glycoconjugates used for the glycoconjugate microarray are shown in Table S3. Gal-4 was labeled with Cy3-N-hydroxysuccinimide ester (GE Healthcare), and unreacted Cy3 was removed using Sephadex G-25 desalting columns (GE Healthcare). Cy3-labeled Gal-4 was then diluted to a final concentration of 10 μg/mL in probing buffer (25 mM Tris-HCl, pH 7.5, 140 mM NaCl, 2.7 mM KCl, 1 mM CaCl_2_, 1 mM MnCl_2_ and 1% Triton X-100). The sample was incubated overnight at 20°C with glycoconjugate microarrays containing 98 glycoconjugates. After incubation, the microarray was washed three times with probing buffer, and fluorescence signals were captured using the Bio-Rex Scan 200 (Rexxam Co. Ltd., Kagawa, Japan).

### 2.9 Tissue staining

All animal experiments were conducted in accordance with the Guidance on the operation of the Animals (Scientific Procedures) Act 1986 and the NIH (National Research Council) Guide for the Care and Use of Laboratory Animals. All procedures were approved by the Animal Research Committee of the University of Tsukuba (protocol numbers: 22–164, 23–113, 24–072). Dorsal skins from naïve wild-type C57BL/6 N mice (male, 8-12 weeks old) were paraffinized as previously described [15]. Skin tissue sections were deparaffinized with xylene and 100, 90, 80, and 70% ethanol. Antigens were retrieved by a 96°C heated citric acid buffer (pH 6.1) for 40 min. To block the non-specific binding, tissue sections were incubated with 1% bovine serum albumin (BSA) (Sigma, MA, USA) in TBS-T (pH 7.4, 0.1% tween20, 50 mM Tris, 150 mM NaCl, 1 mM MgCl_2_, and 1 mM CaCl_2_) for 1 h, followed by incubation with PE-labeled BSA or PE-labeled Gal-4 (10 μg/ml) in the presence or absence of HP-BSA (20 μg/ml) for 2 h at room temperature [20]. Skin mast cells in the section were stained with FITC-labeled avidin (4 μg/ml) (Thermo Fisher Scientific, MA, USA) for 30 min at room temperature, and the nuclei were stained using Prolong™ Gold Antifade Mountant with DAPI (Thermo Fisher Scientific, MA, USA).

### 2.10 Molecular dynamics (MD) simulations-assisted modeling

The coordinates of an HP [repeat of *N*, *O6*-disulfo-glucosamine (SGN) and *O2*-sulfoglucronic acid (IDS)] and the N- and C-terminal CRDs of Gal-4 were obtained from Protein Data Bank (entry codes 1HPN, 5DUV, and 4YM3, respectively). Force field parameters for SGN and IDS were generated by antechamber and parmchk2 tools implemented in the AMBER 19.0 package [21]. As the initial model of the complex, an octasaccharide part in model 1 of the HP structure was fitted to the CRD groove that includes the lactose binding site, by using the PyMOL ver. 2.5 (Schrodinger, LLC). During the fitting procedure, the HP octasaccharide was kept in its helical conformation, and a series of rotational and translational adjustments were applied to position the octasaccharide for optimal fitting into the groove. When necessary, the orientations of sulfate groups were adjusted to avoid steric clashes with the protein. For each CRD, both possible orientations of the HP octasaccharide were examined. When the glycan was released from the protein during the 20 ns MD simulation (see below), a different initial model with an altered position was employed.

The MD calculations were performed by AMBER 19.0 [21] essentially as described previously [22]. Briefly, after minimization, stepwise calculations were performed; 1) equilibration of water and ion molecules at 100 K and a constant pressure of 1 atm, 2) runs at 1 atm with increasing temperature up to 300 K by a step of 100 K, 3) equilibration run at 300 K in the constant volume mode, and 4) production run for 20 ns. The representative structures were selected as the lowest rms deviations (RMSDs) from the average coordinates in the production runs of the last 5 ns (15 ns–20 ns). RMSDs of the glycan non-hydrogen atoms from the representative structure were calculated over 2500 trajectory frames of this period after alignment of the protein moiety.

### 2.11 Sequence alignments

Amino acid sequences of galectins were obtained from the NCBI database (https://www.ncbi.nlm.nih.gov/protein/) with the accession numbers NP_002296 (Galectin-1), NP_006489 (Galectin-2), NP_002297.2 (Galectin-3), NP_006140.1 (Galectin-4), NP_002298.1 (Galectin-7), KAI4085470 (Galectin-8), and KAI4048456 (Galectin-9). The multiple alignment was performed using Clustal Omega [23] at the EBI site (https://www.ebi.ac.uk/jdispatcher/msa/clustalo).

## 3 Results

### 3.1 Generation of GAG microarrays

The GAGs used in this study included HP, CSA, CSC, CSE, CH, and OxCH (Fig. 1A and S1, Table S1). To enable conjugation, thiol (–SH) groups were introduced at the reducing end of each GAG. BSA was then functionalized with N-(11-maleimido-undecanoyloxy)succinimide via its amino groups and subsequently conjugated to GAG-SH. The resulting GAG-BSA conjugates were printed in triplicate onto epoxy-activated glass slides. The amount of biotinylated GAG deposited in each spot was quantified using Cy3-labeled streptavidin (Cy3-SA)(Fig. S2) [15]. Comparable levels of deposition of GAG species in each spot were confirmed by the Cy3-SA signal intensity (Fig. S2).

**Fig. 1:**
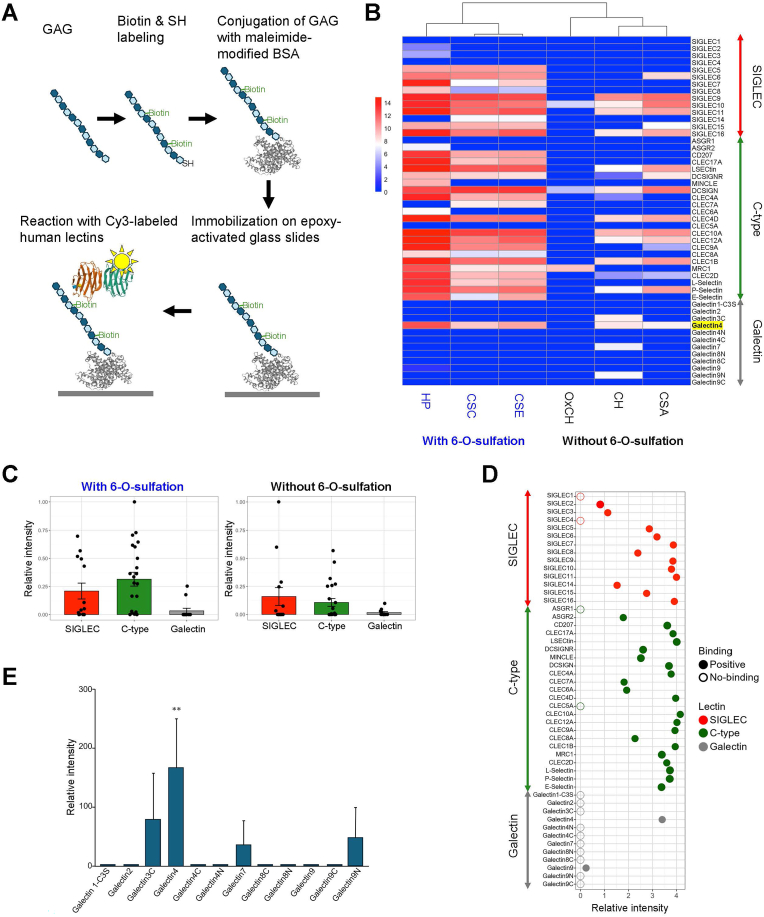
Generation and analysis of GAG microarrays. (A) Schematic overview of the generation and analysis of the GAG microarrays. (B) Heatmap of the binding signals of 49 types of human endogenous lectins to GAGs. A red-to-blue color gradient represents high to low lectin binding intensity. (C) Bar graph comparing the binding of SIGLEC, C-type lectin, and galectin families to GAGs with or without 6-O-sulfation. Values represent the mean binding signals of all lectins within each family for the indicated GAG groups. (D) Dot plot summarizing the binding of SIGLEC, C-type lectin, and galectin family members to 6-O-sulfated GAGs. (E) Statistical comparison of Gal-4 binding to 6-O-sulfated GAGs compared with all other galectin family members. Wilcoxon rank-sum test. Significance: ∗∗∗p < 0.001, ∗∗p < 0.01, ∗p < 0.05, ns = not significant.

### 3.2 Screening of the GAG-binding specificity of endogenous lectins

To evaluate the binding specificity of human endogenous lectins toward GAGs, we analyzed interactions of a comprehensive lectin panel (49 types)—including SIGLECs (14 types), C-type lectins (23 types), and galectins (12 types)—using GAG microarrays (Fig. 1B–Table S2). GAGs containing 6-O-sulfation, such as HP, CSC, and CSE, displayed broad binding across multiple lectin classes, particularly SIGLECs and C-type lectins (Fig. 1B and S1). In contrast, GAGs lacking 6-O-sulfation, including CH, oxCH, and CSA, showed minimal or negligible interactions.

To quantify these family-specific trends, we compared relative binding intensities within each lectin family toward GAGs with or without 6-O-sulfation (Fig. 1C). Although binding strength varied depending on the specific sulfation pattern of individual GAGs, a consistent pattern emerged: SIGLEC and C-type lectin families exhibited both higher binding frequency and greater overall binding intensity than galectins (Fig. 1C). Moreover, these two families showed a clear preference for 6-O-sulfated GAGs over non-sulfated counterparts.

We further performed a systematic comparison of individual lectin binding profiles toward 6-O-sulfated GAGs across the three families (Fig. 1D). Within the SIGLEC family, most members displayed strong binding, except for SIGLEC1 and SIGLEC4, which showed relatively weaker interactions. Similarly, C-type lectins generally demonstrated robust recognition of sulfated GAGs, although ASGR1 and CLEC5A exhibited comparatively low binding activity. In contrast, most galectins showed minimal affinity for 6-O-sulfated GAGs. Notably, galectin-4 (Gal-4) was a clear exception, displaying substantially stronger binding than other galectins (Fig. 1E).

Gal-4 is a tandem-repeat galectin predominantly expressed in epithelial tissues, particularly in the gastrointestinal tract, where it regulates epithelial differentiation, cell adhesion, membrane organization, and barrier integrity [16]. Previous studies have also shown that Gal-4 recognizes sulfated glycans and mediates glycan-dependent cellular interactions [[24], [25], [26]]. These findings suggest that Gal-4 may function as a regulator of cell-surface and extracellular matrix (ECM) glycan signaling. Given that sulfated GAGs are major components of the glycocalyx and ECM, and play critical roles in processes such as cell adhesion, migration, growth factor signaling, and tumor progression, the strong binding activity of Gal-4 identified in our screening highlights its potential functional importance. Accordingly, we selected Gal-4 as a representative galectin for subsequent functional analyses.

### 3.3 Glycan-binding specificity and affinity of Gal-4

The glycan-binding specificity of Gal-4 was further analyzed using glycoconjugate microarrays containing 98 types of glycopolymers and glycoproteins (Fig. 2A, S3, Table S3). Among the 98 glycoconjugates, Gal-4 showed significant binding to HP-BSA, in agreement with the results obtained by GAG microarray analysis (Fig. 1B). Gal-4 also exhibited binding to di-GalNAcβ (GalNAcβ1-4GalNAc), asialo glycophorin (AsialoGP) containing asialo O-glycans, and thyroglobulin containing asialo N-glycans, in agreement with the previous report that Gal-4 binds to βGal and βGalNAc-terminated glycans [27]. Gal-4 bound to HP-BSA in a concentration-dependent manner (Fig. 2B). Treatment with 0.2 M lactose (Gal-4 + Lac) abolished Gal-4 binding to most glycoconjugates, whereas only partial inhibition was observed with HP-BSA. In contrast, 10 μg/mL HP-BSA (Gal-4 + HP) inhibited Gal-4 binding to HP-BSA itself but had little effect on other glycoconjugates. These findings suggest that Gal-4 does not primarily interact with HP via its canonical carbohydrate-recognition domains and instead involves additional binding determinants.

**Fig. 2:**
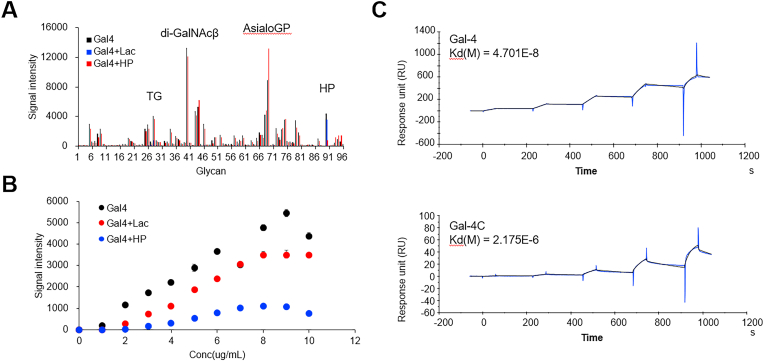
Glycoconjugate microarray analysis of Gal-4. (A) Glycoconjugate microarrays were incubated with Cy3-labeled Gal-4 (10 μg/mL) in the absence (Gal-4) or presence of 0.2 M lactose (Gal-4+Lac) or 10 μg/mL HP-BSA, followed by fluorescence detection using a scanner. Data represent the average ± SD of triplicate spots. Raw microarray data are shown in Fig. S3. (B) Concentration-dependent analysis of the binding of Gal-4 (1-10 μg/mL) to HP in the absence (Gal-4) or presence of 0.2 M lactose (Gal-4+Lac) or 10 μg/mL HP-BSA. Data represent the average ± SD of triplicate spots. (C) Binding affinity of Gal-4 to HP-BSA was analyzed by surface plasmon resonance.

The binding affinity of Gal-4 to HP-BSA was then evaluated using SPR (Fig. 2C). HP-BSA was immobilized on the sensor chip, and interactions were analyzed with varying concentrations of Gal-4. The dissociation constant (Kd) of Gal-4 to HP-BSA was calculated to be 4.70 × 10^−8^ M. Gal-4 consists of tandemly repeated N-terminal (Gal-4N) and C-terminal CRDs (Gal-4C), and their binding affinity to HP-BSA was also evaluated. The Kd of Gal-4C showed a value of 2.175 × 10^−6^ M, indicating a weaker dissociation constant compared to the full-length form. In contrast, the Kd of Gal-4N to HP-BSA was not detected under experimental conditions (Fig. S4).

### 3.4 Gal-4 interacts with HP-positive mast cells

Naïve dermatitis mouse skin was stained with HRP-conjugated Gal-4 and analyzed by multiplex immunostaining (Fig. 3). Gal-4 signals colocalized with mast cells, identified using FITC-conjugated avidin, a mast cell marker [28]. Notably, Gal-4 binding to mast cells was markedly inhibited by 10 μg/mL HP-BSA, indicating competitive inhibition, consistent with the glycoconjugate microarray results (Fig. 2A). These findings demonstrate that Gal-4 specifically binds to HP-positive mast cells.

**Fig. 3:**
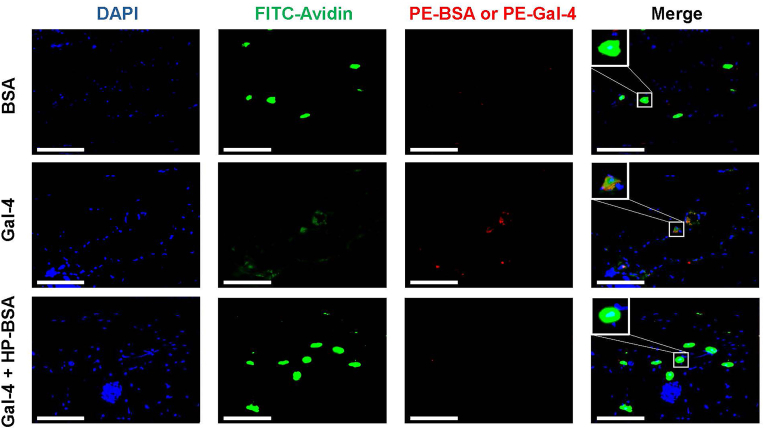
Gal-4 binding to HP-positive mast cells in mouse skin. Mouse skin tissue sections were co-stained with FITC-avidin (green) and PE-Gal-4 or PE-BSA in the presence or absence of HP-BSA. Scale bar: 100 μm.

### 3.5 Structural aspects for Gal-4–HP interactions

We have elucidated the interaction between Gal-4 and HP on the basis of 3D structures. For the two CRDs (Gal-4N and Gal-4C), crystal structures of the complexes with lactose are available [29,30](Fig. 4A). In the surface of these structures, shallow grooves that include lactose-binding sites are observed, in which the coordinate of a HP octasaccharide (a repeat of IDS[α1-4]SGN[α1-4] derived from an NMR-based structural model [32]) can be placed reasonably (Fig. 4B). Then we performed MD simulations for 20 ns, where both orientations of the octasaccharides concerning the reducing and non-reducing ends were examined (Fig. 4C, Fig. S5). The stability of the complex was evaluated by the RMSD values of glycan after alignment of the protein moiety. The value for the Gal-4C model in Fig. 4C was 1.44 ± 0.25 Å, which is comparable to that in the protein moiety (1.28 ± 0.31 Å) and indicates a reasonable stability. For the Gal-4N models in Fig. S5B and D, and the Gal-4C model in Fig. S5F, those of 4.10 ± 1.56, 1.47 ± 0.32, and 2.36 ± 1.10 Å were obtained. They are less stable except for the Gal-4N model in Fig. S5D.

**Fig. 4:**
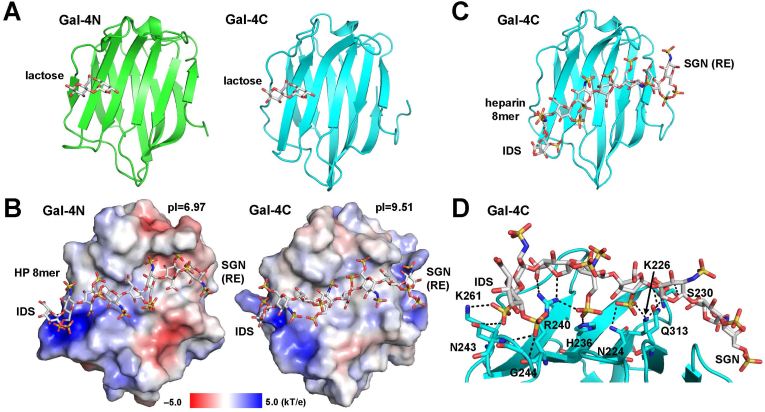
MD-assisted modeling of the Gal-4–HP interaction. (A) Crystal structures of N- and C-terminal CRDs (Gal-4N and Gal-4C) binding lactose. (B) Electrostatic surfaces of CRDs (the left and right panels are for Gal-4N and Gal-4C, respectively) generated by the Adaptive Poisson-Boltzmann Solver (APBS) [31] used via PyMOL ver. 2.5 (Schrodinger, LLC). A HP octasaccharide (IDS[α1-4]SGN[α1-4]IDS[α1-4]SGN[α1-4]IDS[α1-4]SGN[α1-4]IDS[α1-4]SGN[α1]-OH derived from PDB entry 1HPN) was fitted into the groove that includes the lactose binding site, and used as the starting structure of the MD simulation. SGN: *N*, *O6*-disulfo-glucosamine. IDS: *O2*-sulfoglucronic acid. R: reducing end. (C) The representative structure of the MD simulation for the HP bound to Gal-4C. (D) Intermolecular hydrogen bonds observed in the representative structure of the Gal-4C-HP complex (black dashed lines; donor-acceptor distance <3.5 Å).

In the SPR experiments, Gal-4C, but not Gal-4N, showed a significant affinity for HP (Fig. 2). According to the pI value, Gal-4N is neutral (pI = 6.97), and its groove showed basic and acidic halves (left and right halves in Fig. 4B, respectively). In the complex models after MD simulations, interactions between HP and the acidic half are relatively scarce or even absent (Fig. S5B and D). In contrast, Gal-4C is strongly basic (pI = 9.51) and has a groove that is positively charged throughout, making it likely to attract the acidic HP. Therefore, models for this domain likely represent the major interaction modes. During the MD simulation, HP remained stably located in the groove and exhibited significant overlap with the lactose-binding site (Fig. 4A and B), which explains the competitive binding as shown in Fig. 2A and B. In the final models, we observed a number of intermolecular hydrogen-bonding interactions (Fig. 4D, Fig. S5F). Especially, those between basic residues i.e., Arg, Lys, and His, and the negatively charged sulfate or carboxyl groups should be important, forming strong charge-assisted hydrogen bonds [33]. These residues in Gal-4C are K219, K226, H236, R240, and K261, among which the latter four are observed in models with HP in both orientations (Fig. 4 and S5).

## 4 Discussion

To comprehensively assess the GAG-binding capacity of endogenous lectins, we examined the binding profiles of 49 types of endogenous lectins, encompassing SIGLECs, C-type lectins, and galectins, using GAG microarray analysis. Many SIGLECs and C-type lectins exhibited binding activity to sulfated GAGs, such as HP, CSC, and CSE, whereas most galectins showed little or no binding to these GAGs. Among galectins, Gal-4 was a notable exception. Unlike other family members that primarily recognize β-galactoside–containing glycans, Gal-4 displayed a remarkable affinity for GAGs with 6-O-sulfation, particularly HP. This property distinguishes Gal-4 from canonical galectins and suggests a distinct and noncanonical glycan recognition mode.

Gal-4 comprises two tandem CRDs. Gal-4C, which is strongly basic and has a groove that is positively charged throughout, is likely to be the major binding site for the acidic HP (Fig. 4). Four basic residues of Gal-4C, K226, H236, R240, and K261, were observed in models with HP in both orientations (Fig. 4). In addition to the major binding by Gal-4C, HP was also predicted to bind to the groove of Gal-4N containing the basic half in the models (Fig. S5). Among the intermolecular interactions, charge-assisted hydrogen bonds involving basic residues such as K44, R45, R67, K73, and R89 of Gal-4N are likely to contribute to the affinity of the full-length protein, at least to some extent (Figs. S5 and S6). Among the basic residues participating in hydrogen bonding predicted, R45, R67, and R89 of Gal-4N and H236, R240, and K261 of Gal-4C are conserved across galectin CRDs (Fig. S6) [26]. In contrast, K44 and K219 occupy equivalent positions but show only marginal conservation among galectins, and K73 and K226 appear to be Gal-4-specific (Fig. S6). Accordingly, these four residues are plausible determinants of HP binding. Notably, K226 in Gal-4C forms hydrogen bonds with both IDS and SGN in our model (Fig. 4D), suggesting a substantial contribution to binding affinity.

Previous reports demonstrated that Gal-4 binds to glycosphingolipids carrying 3-O-sulfated galactose residues ([3S]Galβ1-3GalNAc) and cholesterol 3-sulfate, which lacks the β-galactoside moiety [26]. The binding of Gal-4 to cholesterol 3-sulfate was inhibited by anionic polysaccharides such as dextran sulfate, KS, CSC, CSA, and HP [26]. Combined with our results, Gal-4 appears to preferentially interact with sulfated glycans, such as sulfated GAGs, sulfated glycolipids, and cholesterol 3-sulfate.

At the cellular level, tissue staining revealed that Gal-4 binds to HP-positive mast cells. Gal-4 may participate in modulating mast cell activity during inflammation. Given that HP becomes accessible on the cell surface after mast cell activation, Gal-4 might preferentially recognize activated mast cells and potentially influence their functional state or microenvironmental interactions.

Gal-4 is predominantly expressed in intestinal epithelial cells and is present intracellularly, on the cell surface, and in extracellular or circulating compartments [16]. In polarized intestinal epithelial cells, it is enriched at the apical brush border membrane and in detergent-resistant membrane/lipid raft fractions, where it contributes to membrane organization and apical glycoprotein trafficking. Gal-4 has also been implicated in epithelial wound repair, innate antibacterial defense, intestinal inflammation, neuronal axon growth and myelination, and tumor progression. In this context, our finding that Gal-4 recognizes sulfated GAGs, particularly HP, suggests that it engages a broader spectrum of endogenous glycans than previously appreciated. Such interactions may contribute to the regulation and organization of cell-surface and extracellular microenvironments. Consistent with this notion, tissue staining demonstrated Gal-4 binding to HP-positive mast cells, supporting the relevance of sulfated GAG recognition in local inflammatory regulation.

While the present study highlights a potential HP-reactive property of Gal-4 distinct from canonical galectin–glycan recognition, Gal-4 could also bind to other endogenous sulfated GAGs with 6-O-sulfation such as heparan sulfate, CSA, and CSE, as partially revealed by GAG microarray analysis (Fig. 1B). Gal-4 may play various physiological roles in homeostasis and disease states through its interactions with these sulfated GAGs. Further studies are essential to understand the roles of Gal-4 binding to sulfated GAGs.

In contrast to Gal-4, other galectins such as Gal-9, Gal-3C, and Gal-7 have been reported to bind to an unsulfated CH tetrasaccharide (GlcAβ1-3GalNAcβ1-4GlcAβ1-3GalNAc) [34]. Consistent with these observations, these galectins also displayed detectable binding to CH in GAG microarray analyses (Fig. 1B). Among C-type lectins, L-selectin (CD62L) is a well-established HS-binding receptor, and endothelial HS is essential for L-selectin-mediated leukocyte recruitment during inflammation [35]. By contrast, although several SIGLECs preferentially recognize sulfated sialylated glycans, direct binding to sulfated GAGs has been reported only for SIGLEC-8, which binds keratan sulfate [36].

Taken together, our results position Gal-4 as a unique galectin exhibiting strong affinity for 6-O-sulfated GAGs, particularly those containing HP. This characteristic distinguishes Gal-4 from other galectins. Further research is needed to elucidate how the interaction between Gal-4 and GAGs contributes to immune regulation and disease development in vivo.

## Funding

This study was supported by joint research funding between AIST and Seikagaku Co., JSPS KAKEN (23K26872, 23H04796), AMED P-PROMOTE (25ama221332h0002), and AMED e-Asia JRP (23jm0210100h0002).

## CRediT authorship contribution statement

**Kanae Sano:** Data curation, Formal analysis, Writing – original draft. **Meri Nagatomo:** Data curation, Formal analysis, Methodology. **Katsunobu Shigematsu:** Data curation, Formal analysis, Methodology. **Kazuhiko Yamasaki:** Data curation, Formal analysis, Writing – original draft. **Dinh Xuan Tuan Anh:** Data curation, Formal analysis. **Akira Shibuya:** Project administration, Resources, Supervision. **Yuya Otsuka:** Project administration, Resources, Writing – review & editing. **Toshikazu Minamisawa:** Project administration, Resources, Supervision, Writing – review & editing. **Hiroaki Tateno:** Conceptualization, Formal analysis, Funding acquisition, Supervision, Writing – original draft.

## Declaration of competing interest

The authors declare the following financial interests/personal relationships which may be considered as potential competing interests:Hiroaki Tateno reports financial support was provided by Seikagaku Corporation. Hiroaki Tateno reports financial support was provided by Japan Society for the Promotion of Science. Hiroaki Tateno reports financial support was provided by Japan Agency for Medical Research and Development. If there are other authors, they declare that they have no known competing financial interests or personal relationships that could have appeared to influence the work reported in this paper.

## Figures and Tables

**figs1:**
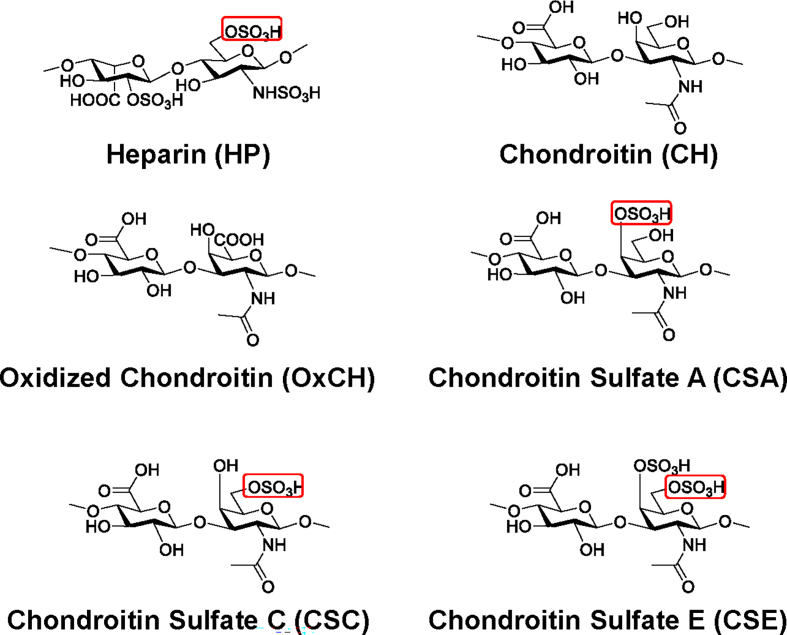


**figs2:**
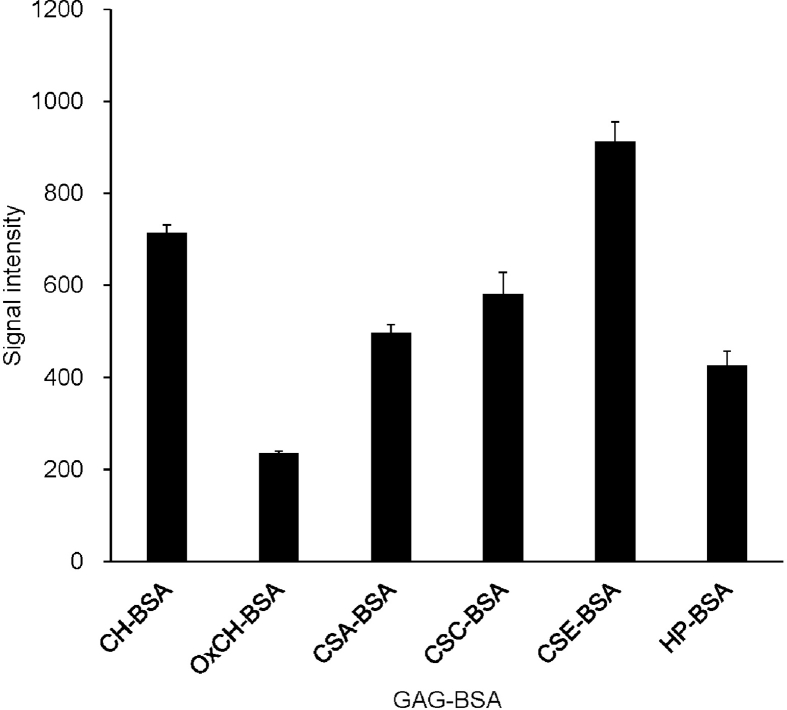


**figs3:**
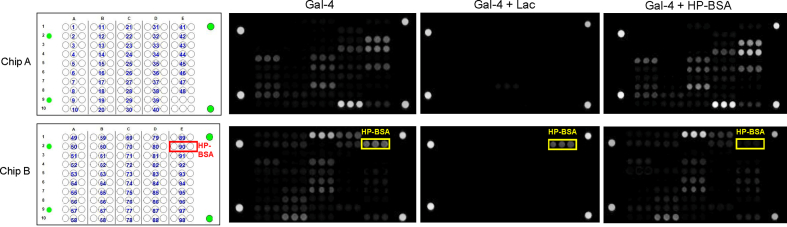


**figs4:**
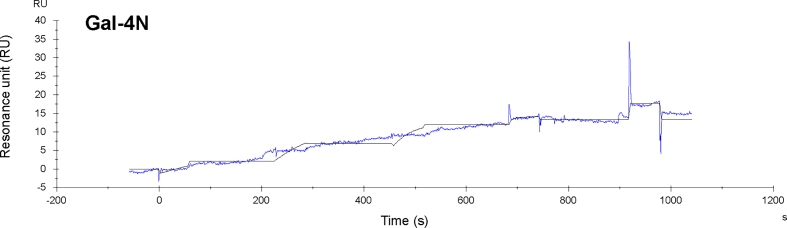


**figs5:**
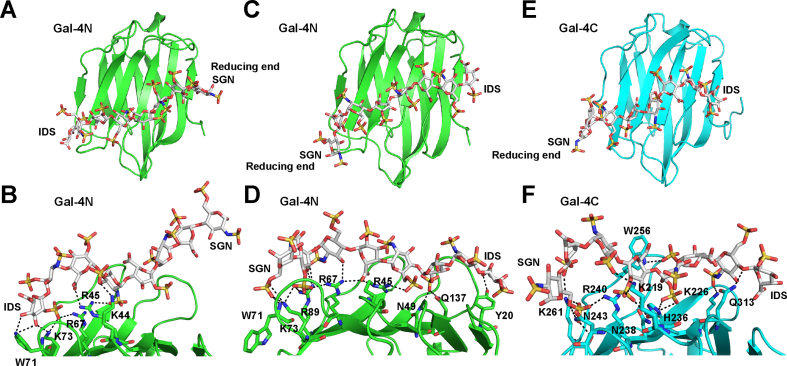


**figs6:**
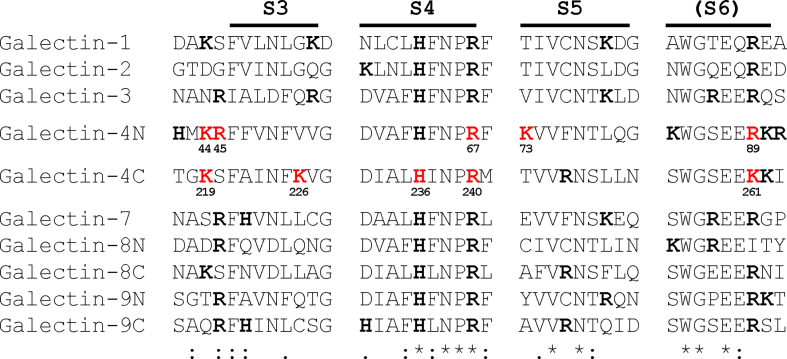


## Data Availability

Data will be made available on request.
